# Characterization of Cellulosic Pulps Isolated from Two Widespread Agricultural Wastes: Cotton and Sunflower Stalks

**DOI:** 10.3390/polym16111594

**Published:** 2024-06-04

**Authors:** M. Dolores La Rubia, Sofía Jurado-Contreras, Francisco Javier Navas-Martos, Ángeles García-Ruiz, Francisca Morillas-Gutiérrez, Alberto J. Moya, Soledad Mateo, José Antonio Rodríguez-Liébana

**Affiliations:** 1 Department of Chemical, Environmental and Materials Engineering, University of Jaén, Campus Las Lagunillas, 23071 Jaén, Spain; 2 University Institute for Research in Olive Grove and Olive Oil (INUO), University of Jaén, Campus Las Lagunillas, 23071 Jaén, Spain; 3 Andaltec Plastic Technological Centre, P.I. Cañada de la Fuente, C/Vilches 34, 23600 Martos, Spain

**Keywords:** lignocellulose, biomass, agricultural wastes, revalorization, cellulose extraction

## Abstract

Globally, huge amounts of cotton and sunflower stalks are generated annually. These wastes are being underutilized since they are mostly burned in the fields. So, in this work, we proposed a three-step method consisting of acid pre-treatment, alkaline hydrolysis, and bleaching for the extraction of cellulose pulps. These pulps were characterized to assess their morpho-structural and thermal properties. The design of experiments and response surface methodology were used for the optimization of the acid pre-treatment in order to achieve maximum removal of non-cellulosic compounds and obtain pulps enriched in cellulose. For cotton stalks, optimal conditions were identified as a reaction time of 190 min, a reaction temperature of 96.2 °C, and an acid (nitric acid) concentration of 6.3%. For sunflower stalks, the optimized time, temperature, and acid concentration were 130 min, 73.8 °C, and 8.7%, respectively. The pulps obtained after bleaching contained more than 90% cellulose. However, special care must be taken during the process, especially in the acid pre-treatment, as it causes the solubilization of a great amount of material. The characterization revealed that the extraction process led to cellulose pulps with around 69–70% crystallinity and thermal stability in the range of 340–350 °C, ready to be used for their conversion into derivatives for industrial applications.

## 1. Introduction

Industry is currently moving towards circular bio-economy models in order to mitigate the effects that the huge greenhouse gas emissions are having on the environment, such as the rise of global temperature. This, coupled with the alarming depletion of fossil resources, has led to extensive research in recent years in search of more eco-friendly alternatives. In this context, lignocellulosic biomass continues to attract global interest for different characteristics, such as its abundant availability, renewability, and carbon-neutral nature. Hence, it is becoming an indispensable feedstock for the synthesis of high-value products such as biofuel, platform chemicals, or biomaterials [1,2,3,4,5]. Lignocellulose is found in secondary plant cell walls. It is mainly comprised of polysaccharides, namely cellulose and hemicelluloses, and an aromatic biopolymer (lignin), which are intimately associated by non-covalent forces and covalent crosslinkages to form a complex and intricately linked structure that provides strength to plant cells [5,6,7]. Also, lower amounts of other compounds such as pectin, extractives, proteins, waxes, or ashes are present in lignocellulosic materials. Chemically, lignocellulosic biomass is, therefore, a mixture of all of these compounds at varying ratios that are largely dependent on the plant species from which they originate.

Based on their origin, lignocellulosic residues can be classified as (i) municipal solid waste, (ii) forest residues, (iii) waste paper, and (iv) agricultural wastes. Millions of tons of the latter are generated annually worldwide by the overproduction of agricultural crops to cover the necessities of modern society. Therefore, among all the available lignocellulosic sources, agricultural wastes have a great potential for revalorization into valuable products because of their high abundance and low cost. Moreover, the revalorization of agricultural wastes may alleviate the challenges of managing their disposal, thus increasing the circularity of agricultural systems. Crop waste is often open-burned in the fields to prepare the land for the next crop. However, burning may impair the quality and fertility of soil, thus affecting long-term crop productivity. In addition, waste burning increases the emissions of greenhouse gases and particulate matter, with direct impacts on climate change, water acidification, air pollution, and human respiratory diseases [5,8,9,10,11].

Cotton (*Gossypium hirsutum*) is one of the most widespread and profitable non-food crops in the world, mainly used for fiber. The cottonseed remaining after ginning is used to produce oil for human consumption and oilseed cake for animal feed. Cotton is grown in subtropical and seasonally dry tropical areas, with most of the world’s production taking place in the northern hemisphere. The main producing countries are India, China, the United States, Brazil, and Pakistan [12]. In the European Union, only 320,000 ha are dedicated to cotton crops, of which 80% correspond to Greece and approximately 20% to Spain (mainly concentrated in the southern region of Andalusia). Although cotton accounts for less than 0.2% of the value of European agricultural production, it is of great regional importance in the two main producing countries [13]. Global cotton production is over 25 Mt, and is expected to reach 28 Mt in 2030 due to the growth in average global yields and, to a lesser extent, to an expansion of the cultivated area. Nonetheless, as most cotton is grown under rain-fed conditions, climate change may undermine the yield growth potential [12].

Sunflower (*Helianthus annus*) is an annual crop that is also widely cultivated all over the world. It is primarily used for the production of oil and confectionary products, but sunflower is also commercialized for animal feeding and as an ornamental plant. In addition, the seeds can be processed into different forms for human food, such as flour, roasted, baked, or boiled as composite functional foods [14]. It is mainly grown in temperate regions, though it tolerates a relatively wide range of environments as compared with other oilseed crops. Historically, Russia and Ukraine have been major producing countries. In 2023/2024, Russia is expected to produce more than 17 Mt, followed by Ukraine (14.5 Mt), the European Union (10.2 Mt), Argentina (4.1 Mt), Turkey (1.5 Mt), and others (7.6 Mt) [15]. Around 700,000 ha are dedicated to sunflower cultivation in Spain, with production (over 800,000 tons) dominated by Castile and Leon, Andalusia, and Castilla-La Mancha [16].

Worldwide, both cotton and sunflower crops generate large amounts of waste, primarily composed of plant stalks. It has been estimated that the cultivation of cotton generates an amount of residue that is equivalent to 3–5 times the weight of the produced fiber [17], whereas 3–7 tons of dry matter per hectare are left in the fields as waste after the harvesting of sunflower crops [18]. To our knowledge, these residues are mostly open-burned on our own cultivation lands, thus contributing to environmental pollution and toxicity to humans and animals. Therefore, due to their lignocellulosic nature, they may present different potential routes for their revalorization into high value materials. For instance, cotton stalks have been processed for the preparation of aerogels [19,20] and nanocellulose, with potential applications for the reinforcement of polymer composites [21,22]. In the case of sunflower, waste stalks have been deemed for the obtainment of a varied range of chemicals and products such as bio-ethanol [23,24], bio-hydrogen and bio-methane [25], bio-surfactant [26], pectin and glucose [27], xylanase enzyme [28], insulating bio-based composites [29], fluting paper [30], and nanofibers [31].

The main aim of this work is the extraction of the cellulosic fraction from cotton and sunflower stalks through a three-step method consisting of acid pre-treatment, alkaline hydrolysis, and chlorine-free bleaching. The process was optimized by using a design of experiments and response surface methodology (RSM) to maximize the removal of hemicellulose and lignin and minimize cellulose solubilization. The cellulose pulps were comprehensively characterized by different techniques to evaluate their morpho-structural and thermal properties. Once isolated, these pulps could be used for the manufacture of different products such as paper, nanofibers and nanocrystals, carboxymethyl cellulose, cellulose acetate, or any other cellulose derivative with application in the industry.

## 2. Materials and Methods

### 2.1. Collection and Conditioning of CS and SFS Wastes

Bulk wastes left in the field after cotton and sunflower cropping were collected from two cultivation areas located near the towns of Jaén and Córdoba, respectively (both in the south of Spain), and stored in big bags made of polypropylene raffia. Sunflower waste was collected in early September 2020, whereas that from cotton cultivation was collected in mid-October 2020. Most of the remaining flowers in the bulk wastes were manually removed in the laboratory, and the remaining stalks, CS and SFS, were ground in a laboratory mill (SM11 model, Retsch Mühle Dietz-Motoren GmbH and Co. KG, Hann, Germany). Unwanted fractions were removed by sieving using a column sieve shaker (Retsch GmbH and Co. KG, Hann, Germany) to obtain CS and SFS fibers with an adequate particle size. For this study, fibers with a particle size in the range of 425–600 µm were selected, as in our previous work on the extraction of cellulose from olive tree pruning [32]. These fibers were finally dried at a mild temperature, between 50 and 60 °C, for several days and stored in sealed plastic bags before further use for cellulose extraction.

### 2.2. Extraction of Cellulose from CS and SFS Fibers

The extraction of cellulose from CS and SFS fibers was conducted by a chemical procedure based on our previous works with other lignocellulosic residues [32,33,34] consisting of an acid pre-treatment (AH), followed by alkaline hydrolysis (BH) and a bleaching reaction (BL). As described below, the experimental conditions for the acid pre-treatment were optimized to maximize the removal of non-cellulosic compounds [32]. All the treatments were carried out in a 1 L glass reactor provided with mechanical stirring at a solid-to-liquid ratio of 1/10 (*w*/*w*) for AH and BH and 1/20 (*w*/*w*) for BL. The residual fibers after the treatments were filtered, washed with abundant water to remove excess reagents until neutral pH, dried at room temperature for several days, and stored in plastic bags. The fibers obtained after each treatment were labelled as X-AH, X-BH, and X-BL (X = CS or SFS). The yields of each reaction (*Yield_AH_*, *Yield_BH_*, and *Yield_BL_*) and that of the whole process (*Yield_WHOLE_*) were calculated as:

(1) $${Yield}_{AH}(\%)=\frac{m_{AH}}{m_{i}}\times 100$$

(2) $${Yield}_{BH}(\%)=\frac{m_{BH}}{m_{AH}}\times 100$$

(3) $${Yield}_{BL}(\%)=\frac{m_{BL}}{m_{BH}}\times 100$$

(4) $${Yield}_{WHOLE}(\%)=\frac{m_{BL}}{m_{i}}\times 100$$

where *m_i_*, *m_AH_*, *m_BH_* and *m_BL_* are the weights (g) of untreated fibers, and after AH, BH, and BL, respectively.

CS and SFS fibers were first subjected to acid pre-treatment with nitric acid (HNO_3_ Puriss. p.a., 65–67%; Honeywell Chemicals, Charlotte, NC, USA). Response Surface Methodology (RSM) was applied to determine the best experimental conditions aimed at removing as much hemicellulose and lignin as possible. The design of experiments (Design-Expert v6.0; Stat-Ease, Inc., Minneapolis, MN, USA) consisted of a face-centered central composite design (FCCD), in which reaction time (*t*), reaction temperature (*T*), and acid concentration (*c*) were the independent variables, while the percentages of removed cellulose (*Y_cel_*), removed hemicellulose (*Y_hem_*), and removed lignin (*Y_lig_*), calculated as the difference between their contents in the original fibers and in the fibers after the AH process, were the response variables. As shown in Table 1, a total of 17 experiments, including three replicates in the central point (AH_1–17_), were performed. The results were analyzed using STATISTICA v8.0 (Statsoft, Tulsa, OK, USA) software. The relationship between the response functions and the coded variables is described by the second-degree polynomial equation:

(5) $$Y=\beta_{0}+\beta_{i}\sum x_{i}+\beta_{ii}\sum x_{i}^{2}+\beta_{ij}\sum x_{i}x_{j}$$

where *Y* is the specific response function, *x_i_* and *x_j_* are independent variables, *β*_0_ is a constant, and *β_i_*, *β_ii_*, and *β_ij_* are linear, quadratic, and interactive coefficients, respectively.

Once the best experimental conditions for AH were selected, the resulting fibers (CS-AH and SFS-AH) were further processed by following an alkaline hydrolysis reaction with an aqueous solution of sodium hydroxide (NaOH 99% purity; ITW Reagents) at a concentration of 6% (*w*/*v*), maintaining a constant temperature of 75 °C for 105 min [32]. Finally, the NaOH-treated fibers were bleached once with a 5% (*v*/*v*) solution of hydrogen peroxide (H_2_O_2_ 30% *v*/*v* AGR; Labkem) at 70 °C. The pH of the bleaching solution was raised to 11–12 by the addition of NaOH, and the mixture with the fibers was stirred for 1 h.

### 2.3. Characterization of CS and SFS Fibers

#### 2.3.1. Chemical Composition

Polysaccharides and lignin contents were determined by duplicate using a process based on a standard method [24,28,35]. Approximately 2 g of each sample were quantitatively hydrolyzed in 10 mL of 72% (*w*/*w*) sulfuric acid (ITW Reagents) for 7 min at 60 °C. The mixture was diluted with 250 mL of distilled water and autoclaved for 45 min at 121 °C and 1.11 atm pressure. After cooling, the liquid fraction was filtered, and the solid residue was washed with distilled water up to reach a total volume of 500 mL in a volumetric flask. The sugar concentration of this hydrolyzate, used for the calculation of cellulose and hemicellulose contents, was determined by injecting 20 µL aliquots (filtered with 0.22 µm nylon syringe filters) into a high-performance liquid chromatograph (Shimadzu Prominence Serie 20, Shimadzu Europe GmbH, Duisburg, Germany) equipped with an automatic injector (SIL-20ACHT), a sugar separation column (7.8 × 300 mm Aminex HPX-87H; Bio-Rad Laboratories Ltd., Hercules, CA, USA), and a refraction index detector (RID-10A). The chromatographic separation was achieved at a column oven temperature of 45 °C with sulfuric acid (5 mM) at a flow rate of 0.6 mL/min as the eluent.

The solid residue after hydrolysis with sulfuric acid was calcined in a muffle furnace at 600 °C for 4 h before the calculation of the acid-insoluble lignin (AIL) with the following equation:

(6) $$AIL(\%)=\frac{m_{solid}-m_{ashes}}{m_{solid}}\times 100$$

being *m_solid_* and *m_ashes_* (g), the weight of the unhydrolyzed solid and that of the ashes after calcination, respectively.

#### 2.3.2. Scanning Electron Microscopy (SEM), Fourier Transformed Infrared (FT-IR) Spectroscopy, X-ray Diffraction (XRD), and Thermogravimetric Analysis (TGA)

The likely morphological changes produced in the fibers as a consequence of the chemical treatment were analyzed by observation of original and bleached CS and SFS fibers in a field emission microscope (FE-SEM Merlin; Carl Zeiss, Oberkochem, Germany) at an acceleration voltage of 15 kV. Previously, the samples were sputtered with gold particles (Q150T ES vacuum sputter coater; Quorum Technologies, Laughton, UK). To identify the main functional groups and assess the changes produced in the biomass after the chemical treatment, FT-IR spectra of untreated fibers and extracted cellulose samples were recorded in a Tensor 27 spectrometer (Bruker, Billerica, MA, USA) in the wavenumber region 4000–400 cm^−1^ at a resolution of 4 cm^−1^ in the attenuated total reflectance (ATR) mode. All the FT-IR spectra were treated (scaled and maximized) and represented with Opus v6.5 software (Bruker). The crystalline properties of the fibers (original and treated) were examined by XRD. Prior to the analysis, the samples were milled and sieved to a particle size below 500 µm. Powdered samples were then analyzed at room temperature in an Empyrean diffractometer (Malvern PANalytical Ltd., Malvern, UK) with a Cu-Kα radiation source in the *2θ* range between 5 and 40° at a rate of 2°/min. The Segal method [36] was applied to calculate the crystallinity index (*CrI*) of the samples from the diffractograms as follows:

(7) $$CrI\left(\%\right)=\frac{I_{200}-I_{am}}{I_{200}}\times 100$$

*I*_200_ in this equation is the maximum intensity of the (200) lattice diffraction peak corresponding to both amorphous and crystalline phases (2*θ* around 22°), and *I_am_* is the intensity scattered exclusively by the amorphous part (2*θ* around 18°) [21,22,37,38,39]. Finally, the thermal properties of CS and SFS fibers and the isolated cellulose pulps were determined in a thermogravimetric analyzer (Q500 equipment; TA Instruments, New Castle, DE, USA). For the analyses, 10–20 mg of powdered samples were weighted in alumina pans and heated at 10 °C/min from room temperature to 800 °C under inert atmosphere (50 mL/min N_2_ flow). The maximum degradation temperatures of the samples (*T_max_*) were retrieved from the differential thermogravimetric (DTG) curves.

## 3. Results

### 3.1. Chemical Composition of CS and SFS Fibers and Cellulose Isolation Process

The chemical composition of the untreated CS and SFS fibers was analyzed before starting the cellulose extraction process. Both CS and SFS are agro-industrial wastes of lignocellulosic nature and, as such, they are formed by a complex and heterogeneous structure in which the three main constituents (cellulose, hemicellulose, and lignin) are intimately associated. As shown in Table 2, both original materials presented a similar composition profile, with minor differences, in which cellulose is the major component. While CS fibers contained 23% cellulose, 14% hemicellulose, and 22% lignin, SFS fibers were composed of 28% cellulose, 15% hemicellulose, and 20% lignin. Our results are lower than those found in previous works for both wastes, especially concerning polysaccharides content. For instance, Zhou et al. (2017) found that CS was composed of 37.1% cellulose, 32.6% hemicellulose, and 25.0% lignin [21]. In another work, the lignin concentration of CS biomass has been also reported to be 25%, but that of polysaccharides was not measured [19]. For hemicellulose and lignin concentrations, Li et al. (2021) obtained similar values to those found in our research (19.82% hemicellulose and 22.24% lignin), but CS content in cellulose was as high as 50.02% [22]. In line with this, Mussana et al. (2018) reported cellulose and lignin concentrations of 42.25% and 20.62%, respectively [20].

Concerning SFS as a whole, Ewulonu et al. (2019) have reported that it contains 41.20% cellulose, followed by 31.30% hemicellulose, and 20.32% lignin [31]. Similar results were obtained by Rudi et al. (2016) with 39.93% cellulose and slightly above 20% lignin [30]. In contrast, other authors found values more close to those in our study, with average concentrations for cellulose, hemicellulose, and lignin of 36.27%, 10.06%, and 18.34% [28], or 35.6%, 17.1%, and 16.7%, respectively [26]. Moreover, the analysis of the SFS polysaccharide composition by different authors revealed that cellulose and hemicellulose constituted 27–34% and 12–20.2% of the total composition, respectively, and that, besides glucose, the sugar composition is primarily represented by xylose, arabinose, and mannose [23,24,25,28]. Other authors have distinguished between the bark and pith parts of SFS since they have different compositions. While SFS pith contains 35–45% cellulose, 5–10% hemicellulose and 3–5% lignin [27], SFS bark has been reported to be composed of 27–45% cellulose, 13.5% hemicellulose, and 14–35% lignin [25,29]. Finally, Rudi et al. (2016) stated that when SFS are de-pithed the concentration of cellulose significantly increases (from 39.93% to 47.37%), whereas that of lignin is not practically affected [30].

As mentioned above, the experimental conditions of the first step of cellulose extraction (namely acid pre-treatment) were optimized by using a FCCD to understand the relationship between the acid-pretreatment factors and the selected responses (Table 1). Depending on these conditions and on the waste itself, yellowing-brownish fibers were obtained after hydrolysis with nitric acid. Table 3 and Table 4 depict the results of *Y_cel_*, *Y_hem,_* and *Y_lig_* for CS and SFS, respectively, as well as the values of *Yield_AH_* for each experiment. As observed, *Yield_AH_* varied in the ranges of 31.50–69.27% and 41.61–84.01 for CS and SFS, respectively, thus suggesting higher recalcitrance for the latter. These values were in general consistent with the hydrolysis conditions for *t*, *T*, and *c*, thus showing a tendency to be reduced at increased *t* and, especially, *T* and *c* due to higher solubilization rates. Under the same design of experiments, we have previously found yields between 30.90% and 80.23% for the hydrolysis of olive tree pruning [32].

*Y_cel_*, *Y_hem,_* and Y_lig_ were the lowest for AH_1_, an experiment that was performed at low *t*, *T*, and *c*. However, the highest removal of the three biopolymers was achieved at intermediate reaction time and temperature, but using the highest concentration of nitric acid (AH_14_), followed by run AH_8_ performed at high levels of the three independent variables. The experimental results in Table 3 and Table 4 were used to model the quadratic regression equations of each response function after eliminating non-significant coefficients. The model equations for *Y_cel_*, *Y_hem,_* and *Y_lig_* for CS (Equations (8)–(10)) and SFS (Equations (11)–(13)) are shown below:

CS fibers:

(8) $$Y_{cel}=39.4-2.67t+5.93T+6.89c+6.83t^{2}-11.36T^{2}$$

(9) $$Y_{hem}=71.1+0.52t+9.03T+12.25c+2.35t^{2}-7.99T^{2}\;+2.10tT-3.73tc$$

(10) $$Y_{lig}=39.2+2.13t+6.62T+21.06c-9.41t^{2}+9.67T^{2}\;+5.32c^{2}-5.23tc+8.89Tc$$

SFS fibers:

(11) $$Y_{cel}=5.77+3.42t+2.52T+4.89c+11.96T^{2}+8.06tc$$

(12) $$Y_{hem}=54.8+4.34t+17.69T+14.17c-7.37t^{2}-5.93c^{2}\;+3.86tT+5.59Tc$$

(13) $$Y_{lig}=64.6+0.61t+8.01T+11.70c-13.42T^{2}-8.18tc\;-3.60Tc$$

**Figure 1:**
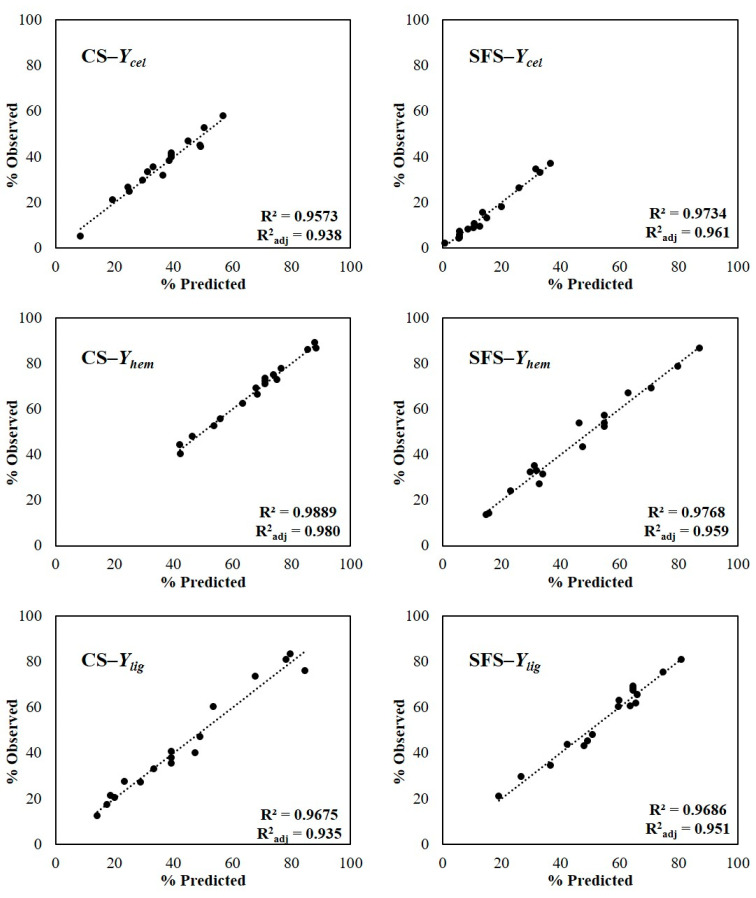
Experimental results for *Y_cel_*, *Y_hem,_* and *Y_lig_* vs. those predicted by the models ((**left**): CS; (**right**): SFS).

The results of the response variables were then fitted to their corresponding model equations to identify the predicted responses, and the accuracy of the model was evaluated by the coefficient of determination (*R*^2^) of the linear regression between observed and predicted responses (Figure 1). As demonstrated in this figure, we can say that the model equations are highly accurate, as confirmed by the high values of *R*^2^ (≥0.95) and adjusted determination coefficients (*R*^2^*_adj_≥* 0.93). Therefore, the model was valid for the prediction of cellulose, hemicellulose, and lignin solubilization rates from both residues within the conditions used.

The effect of either *t*, *T,* or *c* on the response variables is reflected in the linear, quadratic, and interactive coefficients of the model equations. Regarding CS (Equations (8)–(10)), the analysis of these coefficients showed that the three linear terms contributed to enhanced hydrolysis of hemicellulose and lignin, but also that *T* and *c* affected cellulose solubilization as expected [32,34]. These effects were more pronounced for *T* and especially *c*, thus confirming the lower influence of reaction time on the hydrolysis of lignocellulosic biomass found in other studies [32,40]. Therefore, although increased temperature and nitric acid concentration are needed to achieve maximum removal of non-cellulosic compounds, thus contributing to cellulose enrichment, it should be taken into account that too harsh conditions could also lead to undesired hydrolysis of cellulose chains. Similar results were found for SFS fibers (Equations (11)–(13)) though in this case, the lower coefficients of *T* and *c* for *Y_cel_* are in line with the above-referred higher recalcitrance of SFS fibers.

Regarding the quadratic terms, *t*^2^ produced a significant increase of *Y_cel_* and *Y_hem_* and a decrease of *Y_lig_* for CS fibers. Nevertheless, this parameter seemed to have less influence on the hydrolysis of SFS biomass (it only affected *Y_hem_*). *T*^2^ also affected the three response variables for CS hydrolysis by increasing *Y_lig_* and reducing *Y_cel_* and *Y_hem_*, but in the treatments with SFS, it produced a significant increase in cellulose hydrolysis and a significant decrease in lignin hydrolysis. The term *c*^2^ had only a positive influence on CS-*Y_lig_* and a negative effect on SFS-*Y_hem_*.

With respect to the interactions among coefficients, different effects were produced in *Y_hem_* and *Y_lig_*, but they did not affect *Y_cel_* in the case of CS. The term *tc* led to a significant decrease in both *Y_hem_* and *Y_lig_*, more pronounced for the latter. Finally, *tT* and *Tc* caused increased solubilization of hemicellulose and lignin, respectively. With regard to SFS, the parameter *tc* produced an increase in cellulose hydrolysis and a decrease in lignin hydrolysis. In addition, the parameter *Tc* led to increased and decreased *Y_hem_* and *Y_lig_*, respectively, and *tT* only contributed to higher hemicellulose hydrolysis.

The response parameters were optimized to identify the best combination of the independent variables to achieve the targeted outcome, in this case, maximum hydrolysis of hemicellulose and lignin while maintaining relatively low cellulose removal. Our approach identified the optimal conditions at *t* = −0.16 (190 min), *T* = 1.41 (96.2 °C), and *c* = 0.44 (6.3%), and *t* = −0.83 (130 min), *T* = −0.08 (73.8 °C), and *c* = 1.22 (8.7%) for the hydrolysis of CS and SFS fibers, respectively. This was confirmed by performing the acid pre-treatment under these conditions. As illustrated in Table 5, the experimental values of *Y_cel_*, *Y_hem,_* and *Y_lig_* were 32.7%, 72.9, and 88.8% for CS, and 0.78%, 54.3%, and 87.0% for SFS. The values predicted by the quadratic equations were in general slightly lower than those from the experimental confirmation, and the model was less accurate in predicting *Y_cel_* with deviations of 14.6% for CS and 20.5% for SFS. For *Y_hem_* and *Y_lig,_* deviations of the predicted values with respect to the experimental ones between 0.9% and 6.1% were found, thus verifying the validity of the model.

**Figure 2:**
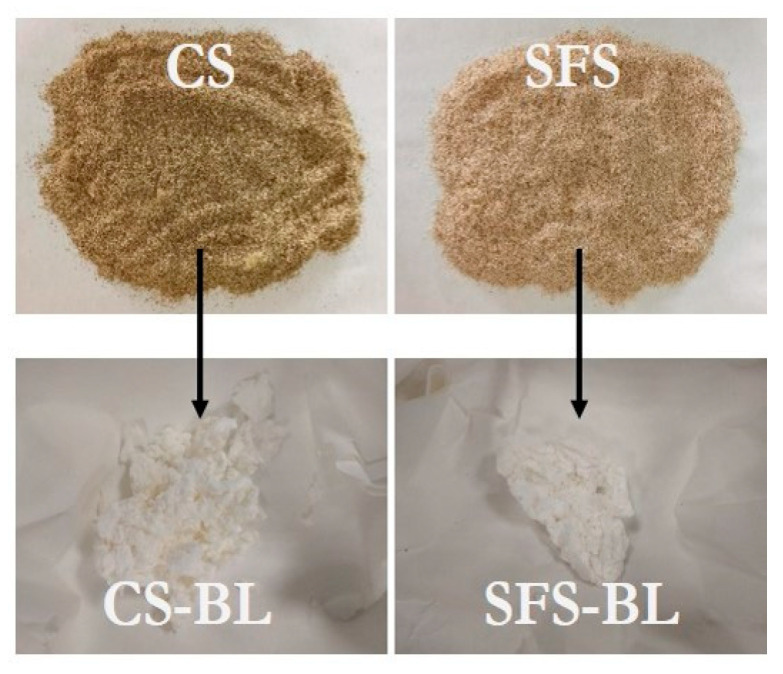
Visual appearance of CS (**left**) and SFS (**right**) fibers before (**top**) and after bleaching (**bottom**).

Under the conditions in Table 5, the concentration of cellulose in CS biomass increased to 52.6%, whereas those of hemicellulose and lignin were approximately halved (Table 2). For SFS, the acid pre-treatment also induced a similar increase in the concentration of cellulose and a decrease in that of non-cellulosic compounds, which was especially intense for lignin (Table 2). After the acid pre-treatment, both CS-AH and SFS-AH fibers (under optimized conditions) were subjected to alkaline hydrolysis and subsequent bleaching, as described in Section 2.2. The pulps obtained at the end of the whole process (CS-BL and SFS-BL) contained above 90% cellulose, while traces of hemicellulose (up to 0.36%) and lignin (up to 2.4%) remained (Table 2). This enrichment in cellulose is in agreement with the color of the fibers that changed from brown (untreated CS and SFS) to white after the treatment (CS-BL and SFS-BL) (Figure 2).

Yield_WHOLE_, calculated as in Equation (4), were 20.1% and 32.0% for CS and SFS wastes, respectively, with the major contribution of acid pre-treatment, especially for CS, followed by alkaline hydrolysis (yields 75.5% for CS-BH and 70.2% for SFS-BH) and bleaching, which reached yields of about 90% for both CS-BL and SFS-BL fibers. Similar results were found for the extraction of cellulose from other widespread lignocellulosic wastes such as sugarcane straw [41], brewer’s spent grains [33], or olive tree pruning [34].

### 3.2. Monitoring of Cellulose Isolation. Characterization of the Fibers by SEM, FT-IR, XRD and TGA

**Figure 3:**
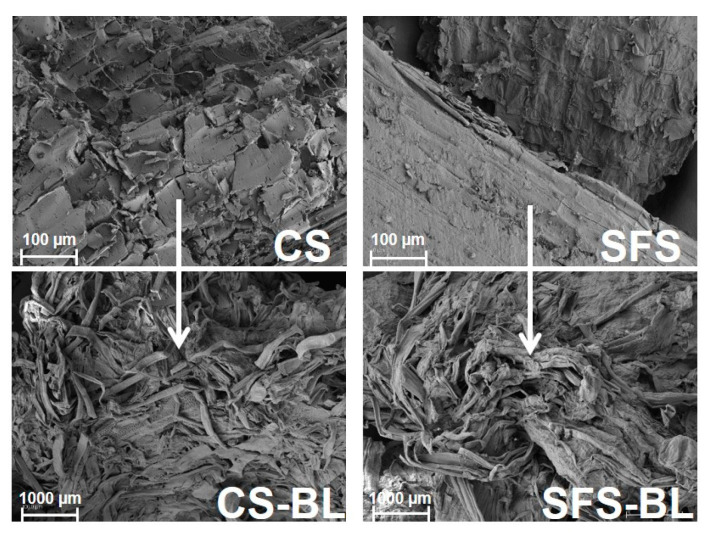
Scanning electron micrographs of CS (**left**) and SFS (**right**) fibers before (**top**) and after bleaching (**bottom**). Scale bars are 100 µm and 1000 µm in the micrographs of untreated and bleached fibers respectively.

The process of cellulose extraction was monitored by analyzing the original CS and SFS fibers and after the chemical treatment through different analytical techniques, namely SEM, FT-IR, XRD, and TGA. Since the chemical treatment led to changes in CS and SFS fibers at the macroscopic level, structural and morphological alterations at the microscopic level in the fibers were also expected. SEM images of the original wastes reflected that both CS and SFS fibers presented a stable structure with a smooth surface typical of lignocellulosic materials (Figure 3 top) [32,33,42,43]. With the progress of the chemical treatment, the lignocellulosic structure was disrupted, and the fiber bundles were disaggregated into loose and almost individualized cellulose fibers (Figure 3 bottom). This indicated that, under the strong conditions of the chemical treatment carried out, massive elimination of the non-cellulosic components that maintain the fibril structure occurred [43]. However, as CS-BL and SFS-BL had residual amounts of hemicellulose and lignin, some of the cellulose microfibers kept bonding together at the end of the process [44].

**Figure 4:**
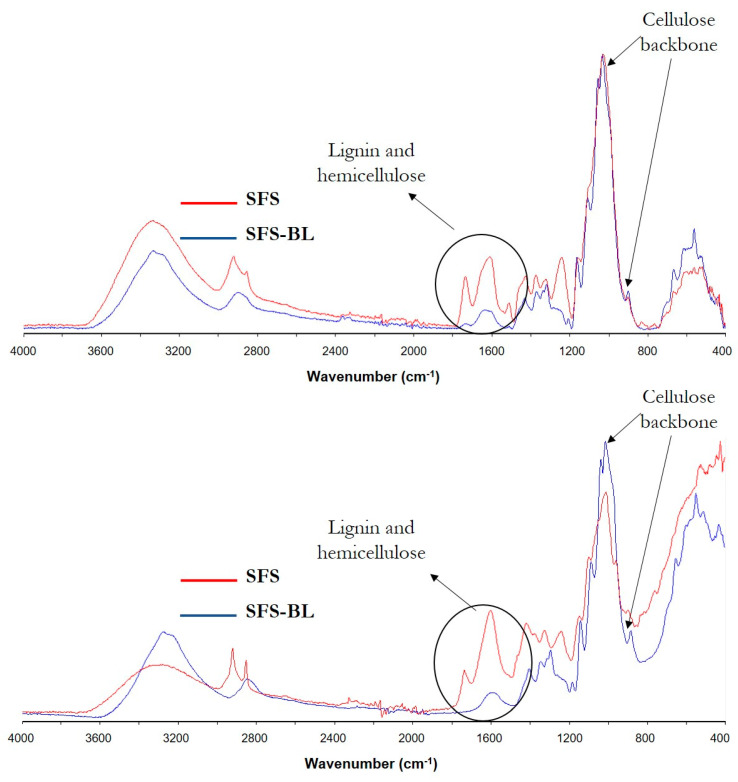
FT−IR spectra of CS (**top**) and SFS (**bottom**) fibers. Red and blue lines represent untreated and bleached fibers, respectively.

The FT-IR spectra of the original fibers and the different cellulose pulps from CS and SFS were also used to evaluate the process of cellulose isolation (Figure 4). Two zones are clearly distinguished in the spectra, in the ranges 800–1800 cm^−1^ and 2800–3600 cm^−1^, as is typical for lignocellulose.

**Figure 5:**
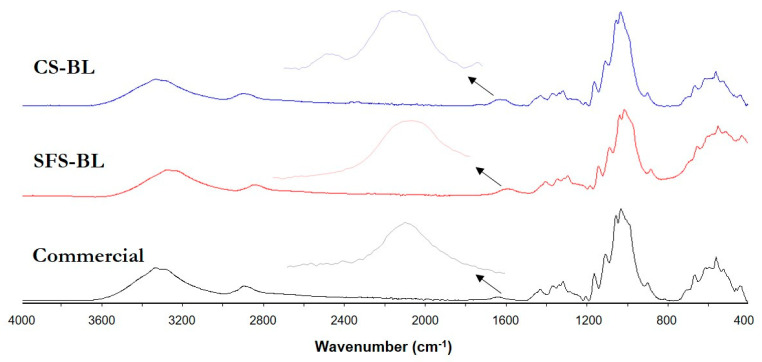
Comparison between the FT−IR spectra of both extracted cellulose pulps and a commercial cellulose sample, and detail of the spectra at the 1800–1500 cm^−1^ region. The spectra were scaled and stacked for comparison.

The broad band in the range of 3600–3000 cm^−1^ represented the stretching vibration of the different -OH groups present in lignocellulose and the retained water [21,37,38,39,45]. The peaks observed in the region 3000–2800 cm^−1^ are associated with the C-H bonds in -CH_3_, -CH_2_ and -O-CH_3_ groups of cellulose, hemicellulose, and lignin [37,38,45,46]. In untreated CS and SFS, this vibration was represented by two well-defined peaks. However, the pattern changed to a lower intensity and smoother band for CS-BL and SFS-BL celluloses. The vibrations at wavenumber 1600–1750 cm^−1^ correspond to the carbonyl region and were related to the presence of esters and other carbonyl-type groups in lignin (carboxylic group of ferulic and p-coumeric acids) and hemicellulose (acetyl and uronic ester groups) structures [21,47,48]. Because of this, the spectra of bleached samples showed reduced absorption in this region. The H-O-H bending of the adsorbed water is also reflected in this region, at a wavenumber of 1640–1630 cm^−1^ [45]. By comparing the spectra of CS-BL and SFS-BL with those of commercial cellulose (microcrystalline powder, Merck Life Science S.L.U., Madrid, Spain) (Figure 5), it is observed that there is a broader band for both the extracted pulps in this region. Hence, the difference can be assumed to be due to the rest of the non-cellulosic components in the bleached samples.

Similarly, the absorptions at 1500–1400 cm^−1^ and 1240–1160 cm^−1^, ascribed to the stretching of C=C linkages of the aromatic rings in lignin [21,47], and to the C−O bonds in cellulose, hemicellulose, and acetyl groups of lignin, respectively [48], had also lower intensity after cellulose extraction. On the other hand, the peaks observed at wavelengths 1100–1020 cm^−1^ and approximately 890 cm^−1^, corresponding to the skeletal vibration of the C-O-C pyranose ring and to the vibration of the glycosidic bonds between cellulose monomers, respectively [37], were better defined in the spectra of CS-BL and SFS-BL. We can therefore say that the typical FT-IR spectrum of lignocellulose observed for both CS and SFS wastes changed to display a pattern that resembled well that of pure cellulose in the case of CS-BL and SFS-BL (Figure 5). This confirmed the effectiveness of the chemical treatment for the isolation of cellulose from CS and SFS, though small amounts of non-cellulosic compounds remained after the chemical treatment.

**Figure 6:**
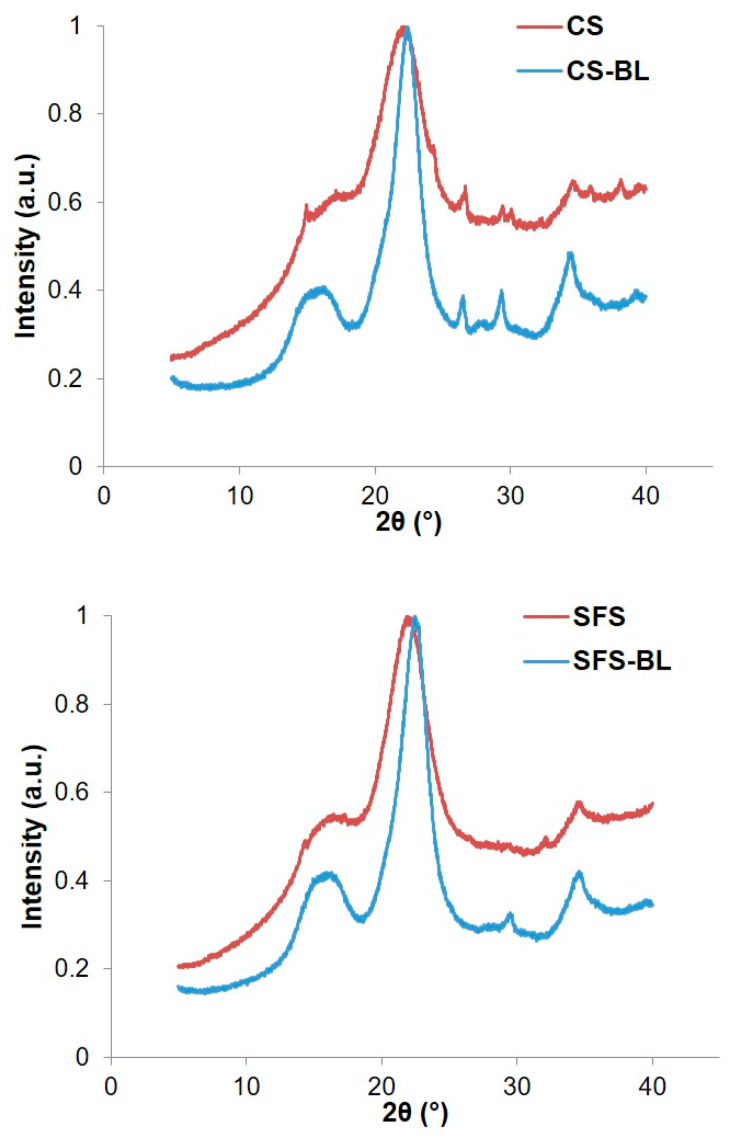
XRD patterns of CS (**top**) and SFS (**bottom**) fibers. Red and blue lines represent untreated and bleached fibers, respectively.

Hemicellulose and lignin are purely amorphous compounds. In contrast, cellulose fibers present both amorphous and crystalline phases in their structure. Therefore, the analysis of the crystalline structure of the samples by XRD before and after the chemical treatment may also be considered a facile method to assess the removal of non-cellulosic compounds by increasing crystallinity. Figure 6 illustrates the XRD patterns of the fibers, both untreated and after the chemical treatment. The original CS and SFS present a principal band at around 2*θ* ≈ 22° (Table 6), which is relatively broad.

This indicated that both wastes have a great amount of amorphous components, namely hemicellulose, lignin, and other non-determined compounds (presumably pectin, waxes, extractive, etc.). After the chemical treatment, three changes were clearly visible in the diffractograms of CS-BL and SFS-BL. From lower to higher *2θ*, an overlapped peak appeared for CS-BL and intensified for SFS-BL at approximately 16.5°. This has been associated with crystal planes with Miller indices of (1–10) and (110) [49]. Other evident changes are the sharpening of the main crystalline peak corresponding to (200) planes (2θ ≈ 22°) and the intensification of the peak at 2θ ≈ 34.5° related to (004) planes, which may also be due to the preferred orientation of crystals. These features are characteristics of cellulose type I [38,44,49], which confirms the massive elimination of amorphous components. Nevertheless, the similarity in the curves for untreated and treated fibers suggests that the chemical treatment did not affect the crystalline structure of the original materials.

Table 6 shows the values of *I_am_* and *I*_200_ for each type of fiber, as well as those for the calculated *CrI*. It was observed that SFS had a higher *CrI* than CS, as expected by its higher cellulose content (Table 2). Other authors have found *CrI* values of 43.3% for CS [20] and 46.8% for SFS [31]. With the treatment, the *CrI* of the CS-BL and SFS-BL cellulose pulps increased to over 69%. It is important to note that the Segal method was developed for native cellulose, so its applicability for the calculation of *CrI* in raw biomass may be controversial. For raw biomass, containing cellulose and an amorphous matrix, *CrI* represents the volume fraction of crystalline cellulose within the given sample. In other words, the determination of *CrI* by XRD can be associated with changes in the volume fraction of cellulose as well as the crystallinity of the sample [50]. Hence, the apparent *CrI* increase observed after hydrolysis is related to the removal of amorphous components rather than to the increase in cellulose crystallinity itself [51]. However, partial destruction of the amorphous regions in the cellulose chains, especially during acid pre-treatment, may also occur [43].

**Figure 7:**
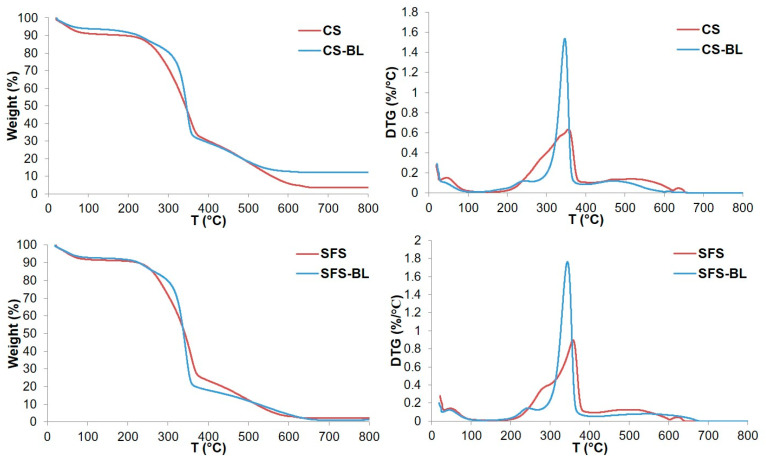
TGA (**left**) and DTG (**right**) curves of CS (**top**) and SFS (**bottom**) fibers. Red and blue lines represent untreated and bleached fibers, respectively.

The analysis of the thermal stability of the fibers (Figure 7) revealed initial weight loss up to 150 °C for all the samples, with values of 9.6% for original CS, 8.6% for original SFS, and 6.7% and 7.5% for bleached CS-BL and SFS-BL, respectively. This initial weight loss has been reported to be related only to the desorption and evaporation of retained water and, hence, to the hydrophilic character of lignocellulosic fibers [20,43]. However, it should also consider the contribution of other volatiles different from water, such as low-molecular weight compounds and extractives [31,44]. This is consistent with the fact that untreated CS and SFS exhibited higher initial weight loss than their corresponding cellulose counterparts, as found previously [34], since the extractives and low-molecular-weight compounds are removed with the chemical treatment. Following this initial loss, rapid decomposition of both untreated and bleached fibers occurred up to approximately 400 °C. This pattern is typical of lignocellulosic materials [20,31,32,38,44] and is due to the simultaneous decomposition of hemicellulose, cellulose, and lignin in this temperature range [52]. Therefore, the shape and height of the peaks in the DTG curves will be dependent on the specific composition of the fibers. The thermograms of the original CS and SFS showed a broad degradation event with discrete peaks at 356 °C and 358 °C, respectively, as observed in the DTG curves. Slightly higher and lower *T_max_* values have been previously reported for CS (373 °C [20]) and SFS (349 °C [31]), respectively. Furthermore, the curve of SFS displayed a shoulder before reaching 300 °C, probably representing the degradation of hemicellulose [20]. After bleaching, the broad bands observed for CS and SFS converted into sharp peaks, with *T_max_* values of 347 °C for CS-BL and 344 °C for SFS-BL, due to cellulose enrichment. This lower *T_max_* value of the bleached fibers with respect to the untreated ones contradicts previous findings, since bleaching normally induces higher thermal stability [32,33,43,44]. Nevertheless, it is worthy to note that, at the *T_max_*, a higher amount of material was decomposed for original fibers (58% for CS and 63% for SFS) than for bleached pulps (52% and 58% for CS-BL and SFS-BL, respectively).

## 4. Conclusions

Cotton and sunflower crops are widely cultivated around the world, and, therefore, they present great potential to be revalorized into high value products for industrial applications. According to the results presented above, it can be concluded that the cellulosic fractions from both CS and SFS wastes were successfully isolated by following a process consisting of an acid pre-treatment, alkaline hydrolysis, and a single-step H_2_O_2_ bleaching. The conditions of the acid pre-treatment were optimized using RSM methodology to achieve maximum removal of non-cellulosic compounds, and the proposed model accurately predicted the hydrolysis of cellulose, hemicellulose, and lignin. However, some precautions should be taken since harsh conditions, especially high temperatures and acid concentrations, could excessively damage cellulose chains. At the end of the whole chemical treatment, both types of fibers were composed of over 90% cellulose and only traces of hemicellulose and lignin.

The analysis of the samples by SEM revealed that the chemical treatment produced a breakage of the typical fibril structure of lignocellulose into loose fibers. As a consequence, the initial smooth surface became rough after bleaching. FT-IR spectroscopy confirmed the isolation of cellulose by a reduction in the signals of the peaks of the characteristics of hemicellulose and lignin and the intensification of those related to the cellulose backbone. Although the crystalline structure of original CS and SFS materials was apparently not affected during the treatment, the crystallinity of the bleached fibers was increased (*CrI* around 70% for both CS-BL and SFS-BL) as compared with that of raw CS (*CrI* = 39.6%) and SFS (*CrI* = 47.4%) due to the elimination of their non-cellulosic components and to the destruction of some amorphous parts in the cellulose structure. The thermal analysis also corroborated the cellulose enrichment, since the decomposition pattern evolved from a broad band for original CS and SFS to a single and sharp peak corresponding to pure cellulose for CS-BL and SFS-BL. Unexpectedly, the fibers showed a slight reduction in their thermal stability after bleaching. Nevertheless, the analysis of the TGA curves revealed that, at the maximum degradation temperature, higher amounts of material remained in the bleached samples as compared with the original ones.

## Figures and Tables

**Table 1:** Coded and real values of the independent variables of the DOE performed for the AH process: *t* reaction time; *T* reaction temperature; *c* acid concentration.

	Coded Values			Real Values		
RUN	*t*	*T*	*c*	*t* (min)	*T* (°C)	*c* (% *w*/*v*)
AH_1_	−1	−1	−1	120	60	2
AH_2_	1	−1	−1	240	60	2
AH_3_	−1	1	−1	120	90	2
AH_4_	1	1	−1	240	90	2
AH_5_	−1	−1	1	120	60	8
AH_6_	1	−1	1	240	60	8
AH_7_	−1	1	1	120	90	8
AH_8_	1	1	1	240	90	8
AH_9_	−1.41	0	0	95.4	75	5
AH_10_	1.41	0	0	264.6	75	5
AH_11_	0	−1.41	0	180	53.85	5
AH_12_	0	1.41	0	180	96.15	5
AH_13_	0	0	−1.41	180	75	0.77
AH_14_	0	0	1.41	180	75	9.23
AH_15_	0	0	0	180	75	5
AH_16_	0	0	0	180	75	5
AH_17_	0	0	0	180	75	5

**Table 2:** Chemical composition of original CS and SFS wastes, and after each stage of treatment. Average values ± Standard Deviation.

Waste Biomass	Cellulose (%)	Hemicellulose (%)	Lignin (AIL; %)	Yield (%)
CS	23.1 ± 0.2	14.2 ± 0.4	21.9 ± 0.1	—
CS-AH ^1^	52.6 ± 1.9	7.5 ± 1.7	11.1 ± 2.5	29.1
CS-BH	75.7 ± 3.8	4.3 ± 0.2	7.8 ± 0.8	75.5
CS-BL	90.6 ± 0.6	0.36 ± 0.06	2.4 ± 0.1	91.7
				Yied_WHOLE_ 20.1
SFS	28.0 ± 0.5	14.7 ± 0.5	20.2 ± 0.4	—
SFS-AH ^1^	50.3 ± 1.7	12.3 ± 1.3	4.7 ± 1.1	51.5
SFS-BH	74.0 ± 1.8	7.5 ± 0.4	5.7 ± 0.2	70.2
SFS-BL	90.8 ± 1.2	0.59 ± 0.09	0.27 ± 0.01	88.6
				Yied_WHOLE_ 32.0

^1^ Under optimized conditions.

**Table 3:** Results for the acid pre-treatment of CS fibers. *Y_cel_* (%), *Y_hem_* (%), *Y_lig_* (%), and *Yield_AH_* are the percentages of hydrolyzed cellulose, hemicellulose, and lignin and the yield of each experiment, respectively.

RUN	*t* (min)	*T* (°C)	*c* (% *w*/*v*)	*Y_cel_* (%)	*Y_hem_* (%)	*Y_lig_* (%)	*Yield_AH_*
AH_1_	120	60	2	26.7	44.1	21.3	69.9
AH_2_	240	60	2	21.1	47.9	32.9	53.0
AH_3_	120	90	2	31.7	55.6	12.4	61.4
AH_4_	240	90	2	33.3	66.3	27.2	54.1
AH_5_	120	60	8	38.2	75.0	60.4	50.4
AH_6_	240	60	8	35.5	62.4	40.0	54.9
AH_7_	120	90	8	52.8	88.9	75.9	32.8
AH_8_	240	90	8	46.9	86.1	81.0	31.7
AH_9_	95.4	75	5	58.0	72.7	17.3	57.9
AH_10_	264.6	75	5	44.4	77.7	27.6	50.2
AH_11_	180	53.85	5	5.2	40.3	47.1	67.6
AH_12_	180	96.15	5	24.9	69.0	73.7	31.5
AH_13_	180	75	0.77	29.6	52.5	20.3	69.4
AH_14_	180	75	9.23	45.1	86.6	83.2	33.0
AH_15_	180	75	5	39.9	71.1	40.6	54.1
AH_16_	180	75	5	41.8	73.3	35.3	54.9
AH_17_	180	75	5	40.8	72.2	38.0	56.6

**Table 4:** Results for the acid pre-treatment of SFS fibers. *Y_cel_* (%), *Y_hem_* (%), *Y_lig_* (%), and *Yield_AH_* are the percentages of hydrolyzed cellulose, hemicellulose, and lignin and the yield of each experiment, respectively.

RUN	*t* (min)	*T* (°C)	*c* (% *w*/*v*)	*Y_cel_* (%)	*Y_hem_* (%)	*Y_lig_* (%)
AH_1_	120	60	2	26.7	44.1	21.3
AH_2_	240	60	2	21.1	47.9	32.9
AH_3_	120	90	2	31.7	55.6	12.4
AH_4_	240	90	2	33.3	66.3	27.2
AH_5_	120	60	8	38.2	75.0	60.4
AH_6_	240	60	8	35.5	62.4	40.0
AH_7_	120	90	8	52.8	88.9	75.9
AH_8_	240	90	8	46.9	86.1	81.0
AH_9_	95.4	75	5	58.0	72.7	17.3
AH_10_	264.6	75	5	44.4	77.7	27.6
AH_11_	180	53.85	5	5.2	40.3	47.1
AH_12_	180	96.15	5	24.9	69.0	73.7
AH_13_	180	75	0.77	29.6	52.5	20.3
AH_14_	180	75	9.23	45.1	86.6	83.2
AH_15_	180	75	5	39.9	71.1	40.6
AH_16_	180	75	5	41.8	73.3	35.3
AH_17_	180	75	5	40.8	72.2	38.0

**Table 5:** Validation of the model for the optimization of acid pre-treatment.

	Experimental Conditions			Experimental Values			Predicted Values		
	*t* (min)	*T* (°C)	*c* (%)	*Y_cel_* (%)	*Y_hem_* (%)	*Y_lig_* (%)	*Y_cel_* (%)	*Y_hem_* (%)	*Y_lig_* (%)
CS	190	96.2	6.3	32.72	72.90	88.78	27.95	73.69	83.35
SFS	130	73.8	8.7	0.78	54.25	87.04	0.62	52.88	86.25

**Table 6:** XRD analysis of CS and SFS samples.

Type of Fiber	Amorphous Fraction		Crystalline Fraction		*CrI* (%)
	2*θ* (°)	*I_am_*	2*θ* (°)	*I*_200_	
CS	17.8	26,435	22.0	43,776	39.6
CS-BL	18.1	6990	22.3	22,597	69.1
SFS	17.5	27,079	21.9	51,527	47.4
SFS-BL	18.6	9136	22.4	30,019	69.6

## Data Availability

The raw data supporting the conclusions of this article will be made available by the authors on request.

## References

[1] Wang F., Ouyang D., Zhou Z., Page S.J., Liu D., Zhao X. (2021). Lignocellulosic biomass as sustainable feedstock and materials for power generation and energy storage. J. Energy Chem..

[2] Chandel A.K., Garlapati V.K., Singh A.K., Fernandes Antunes F.A., da Silva S.S. (2018). The path forward for lignocellulose biorefineries: Bottlenecks, solutions, and perspective on commercialization. Bioresour. Technol..

[3] Singh N., Singhania R.R., Nigam P.S., Dong C.D., Patel A.K., Puri M. (2022). Global status of lignocellulosic biorefinery: Challenges and perspectives. Bioresour. Technol..

[4] Grace Karp S., Bittencourt Sydney E., Lorenci Woiciechowski A., Junior Letti L.A., de Carvalho J.C., Zevallos Torres L.A., Sprotte Kumlehn G., de Souza Candeo E., Soccol C.R., Pandey A., Dayal Tyagi R., Varjani S. (2021). Lignocellulosic biorefinery for value-added products: The emerging bioeconomy. Biomass, Biofuels, Biochemicals. Circular Bioeconomy—Current Developments and Future Outlook.

[5] Ojo A.O. (2023). An overview of lignocellulose and its biotechnological importance in high-value product production. Fermentation.

[6] Zoghlami A., Paës G. (2019). Lignocellulosic biomass: Understanding recalcitrance and predicting hydrolysis. Fermentation.

[7] Dean Smith M., Dean Smith M. (2019). An abbreviated historical and structural introduction to lignocellulose. Understanding Lignocellulose: Synergistic Computational and Analytic Methods.

[8] Kumar P., Joshi L., Nautiyal S., Rao K., Kaechele H., Raju K., Schaldach R. (2013). Pollution caused by agricultural waste burning and possible alternate uses of crop stubble: A case study of Punjab. Knowledge Systems of Societies for Adaptation and Mitigation of Impacts of Climate Change.

[9] de Zárate I.O., Ezcurra A., Lacaux J.P., Van Dinh P., de Argandoña J.D. (2005). Pollution by cereal waste burning in Spain. Atmos. Res..

[10] Shi T., Liu Y., Zhang L., Hao L., Gao Z. (2014). Burning in agricultural landscapes: An emerging natural and human issue in China. Landsc. Ecol..

[11] Zhang L., Liu Y., Hao L. (2016). Contributions of open crop straw burning emissions to PM_2.5_ concentrations in China. Environ. Res. Lett..

[12] OECD/FAO (2023). OECD-FAO Agricultural Outlook 2023–2032.

[13] Cotton. https://agriculture.ec.europa.eu/farming/crop-productions-and-plant-based-products/cotton_en.

[14] Adeleke B.S., Babalola O.O. (2020). Oilseed crop sunflower (*Helianthus annus*) as a source of food: Nutritional and health benefits. Food Sci. Nutr..

[15] Production Volume of Sunflower Seed in Major Producer Countries in 2023/2024. https://www.statista.com/statistics/263928/production-of-sunflower-seed-since-2000-by-major-countries/.

[16] OLEAGINOSAS EN ESPAÑA, EN LA UE-27 Y EN EL MUNDO (Girasol, Colza y Soja). https://www.mapa.gob.es/es/agricultura/temas/producciones-agricolas/anexooleaginosas2021_tcm30-563740.pdf.

[17] Silanikove N., Levanon D. (1986). Cotton straw: Composition, variability and effect of anaerobic preservation. Biomass.

[18] Marechal V., Rigal L. (1999). Characterization of by-products of sunflower culture—Commercial applications for stalks and heads. Ind. Crop. Prod..

[19] Rahbar Shamskar K., Heidari H., Rashidi A. (2016). Preparation and evaluation of nanocrystalline cellulose aerogels from raw cotton and cotton stalk. Ind. Crop. Prod..

[20] Mussana H., Yang X., Tessima M., Han F., Igbal N., Liu L. (2018). Preparation of lignocellulose aerogels from cotton stalks in the ionic liquid-based co-solvent system. Ind. Crop. Prod..

[21] Zhou L., He H., Jiang C., Ma L., Yu P. (2017). Cellulose nanocrystals from cotton stalk for reinforcement of poly(vinyl alcohol) composites. Cellul. Chem. Technol..

[22] Li M., He B., Chen Y., Zhao L. (2021). Physicochemical properties of nanocellulose isolated from cotton stalk waste. ACS Omega.

[23] Ruiz E., Cara C., Ballesteros M., Manzanares P., Ballesteros I., Castro E., McMillan J.D., Adney W.S., Mielenz J.R., Klasson K.T. (2006). Ethanol production from pretreated olive tree wood and sunflower stalks by an SSF process. Twenty-Seventh Symposium on Biotechnology for Fuels and Chemicals. ABAB Symposium.

[24] Nargotra P., Sharma V., Gupta M., Kour S., Kumar Bajaj B. (2018). Application of ionic liquid and alkali pretreatment for enhancing saccharification of sunflower stalk biomass for potential biofuel-ethanol production. Bioresour. Technol..

[25] Monlau F., Sambusiti C., Barakat A., Guo X.M., Latrille E., Trably E., Steyer J.P., Carrere H. (2012). Predictive models of biohydrogen and biomethane production based on the compositional and structural features of lignocellulosic materials. Environ. Sci. Technol..

[26] Passos Santos B.L., Santos Jesus M., Mata F., Oliveira Santos Prado A.A., Monteiro Vieira I.M., Castor Ramos L., López J.A., Vaz-Velho M., Santos Ruzene D., Pereira Silva D. (2023). Use of agro-industrial waste for biosurfactant production: A comparative study of hemicellulosic liquors from corncobs and sunflower stalks. Sustainability.

[27] Zhang Q., Cheng L., Ma X., Zhou X., Xu Y. (2021). Revalorization of sunflower stalk pith as feedstock for the coproduction of pectin and glucose using a two-step dilute acid pretreatment process. Biotechnol. Biofuels.

[28] de Souza J.B., Michelin M., Amâncio F.L.R., Vital Brazil O.A., Polizeli M.d.L.T.M., Ruzene D.S., Silva D.P., Mendonça M.d.C., López J.A. (2020). Sunflower stalk as a carbon source inductive for fungal xylanase production. Ind. Crop. Prod..

[29] Mati-Bouche N., De Baynast H., Lebert A., Sun S., Sacristan Lopez-Mingo C.J., Leclaire P., Michaud P. (2014). Mechanical, thermal and acoustical characterizations of an insulating bio-based composite made from sunflower stalks particles and chitosan. Ind. Crop. Prod..

[30] Rudi H., Resalati H., Behrooz Eshkiki R., Kermanian H. (2016). Sunflower stalk neutral sulfite semi-chemical pulp: An alternative fiber source for production of fluting paper. J. Clean. Prod..

[31] Ewulonu C.M., Liu X., Wu M., Huang Y. (2019). Ultrasound-assisted mild sulphuric acid ball milling preparation of lignocellulose nanofibers (LCNFs) from sunflower stalks (SFS). Cellulose.

[32] Rodríguez-Liébana J.A., Navas-Martos F.J., Jurado-Contreras S., Morillas-Gutiérrez F., Mateo S., Moya A.J., La Rubia M.D. (2024). Manufacture and characterisation of polylactic acid biocomposites with high-purity cellulose isolated from olive pruning waste. J. Reinf. Plast. Compos..

[33] Camacho-Núñez L., Jurado-Contreras S., La Rubia M.D., Navas-Martos F.J., Rodríguez-Liébana J.A. (2023). Cellulose-Based Upcycling of Brewer’s Spent Grains: Extraction and Acetylation. J. Polym. Environ..

[34] Rodríguez-Liébana J.A., Robles-Solano E., Jurado-Contreras S., Morillas-Gutiérrez F., Moya A.J., Mateo S., Navas-Martos F.J., La Rubia M.D. (2024). Production and characterization of cellulose acetate using olive tree pruning biomass as feedstock. Biofuels Bioprod. Bioref..

[35] Sluiter A., Hames B., Ruiz R., Scarlata C., Sluiter J., Templeton D., Crocker D. (2008). Determination of Structural Carbohydrates and Lignin in Biomass.

[36] Segal L., Creely J.J., Martin A.E., Conrad C.M. (1959). An empirical method for estimating the degree of crystallinity of native cellulose using X-Ray diffractometer. Text. Res. J..

[37] Kassab Z., Abdellaoui Y., Hamid Salim M., Bouhfid R., El Kacem Qaiss A., El Achaby M. (2020). Micro- and nano-celluloses derived from hemp stalks and their effects as polymer reinforcing materials. Carbohydr. Polym..

[38] Raza M., Abu-Jdayil B., Banat F., Al-Marzouqi A.H. (2022). Isolation and characterization of cellulose nanocrystals from date palm waste. ACS Omega.

[39] Rubiyah M.H., Melethil K., Varghese S., Kurian M., Babu S., Jojo L., Thomas B. (2023). Isolation and characterization of cellulose nanofibrils from agro-biomass of Jackfruit (*Artocarpus heterophyllus*) rind, using a soft and benign acid hydrolysis. Carbohydr. Polym. Technol. Appl..

[40] Jiménez L., Pérez I., de la Torre M.J., García J.C. (1999). The effect of processing variables on the soda pulping of olive tree wood. Bioresour. Technol..

[41] Candido R.G., Conçalves A.R. (2016). Synthesis of cellulose acetate and carboxymethylcellulose from sugarcane straw. Carbohydr. Polym..

[42] Mussatto S.I., Rocha G.J.M., Roberto I.C. (2008). Hydrogen peroxide bleaching of cellulose pulps obtained from brewer’s spent grain. Cellulose.

[43] Johar N., Ahmad I., Dufresne A. (2012). Extraction, preparation and characterization of cellulose fibres and nanocrystals from rice husk. Ind. Crop. Prod..

[44] Andrade Alves J.A., Lisboa dos Santos M.D., Cintra Morais C., Ramirez Ascheri J.L., Signini R., dos Santos D.M., Cavalcante Bastos S.M., Ramirez Ascheri D.P. (2019). Sorghum straw: Pulping and bleaching process optimization and synthesis of cellulose acetate. Int. J. Biol. Macromol..

[45] Fei P., Liao L., Cheng B., Song J. (2017). Quantitative analysis of cellulose acetate with a high degree of substitution by FTIR and its application. Anal. Methods.

[46] Sun X.F., Jing Z., Fowler P., Wu Y., Rajaratnam M. (2011). Structural characterization and isolation of lignin and hemicelluloses from barley straw. Ind. Crop. Prod..

[47] Dos Santos D.M., De Lacerda Bukzem A., Ascheri D.P.R., Signini R., De Aquino G.L.B. (2015). Microwave-assisted carboxymethylation of cellulose extracted from brewer´s spent grain. Carbohydr. Polym..

[48] Elyamine A.M., Moussa M.G., Afzal J., Rana M.S., Imran M., Zhao X., Hu C.X. (2019). Modified rice straw enhanced cadmium (II) immobilization in soil and promoted the degradation of phenantrene in co-contaminated soil. Int. J. Mol. Sci..

[49] French A.D. (2014). Idealized powder diffraction patterns for cellulose polymorphs. Cellulose.

[50] Harris D., DeBolt S. (2008). Relative crystallinity of plant biomass: Studies on assembly, adaptation and acclimation. PLoS ONE.

[51] Puris V.P. (1984). Effect of crystallinity and degree of polymerization of cellulose on enzymatic saccharification. Biotechnol. Bioeng..

[52] Yang H., Yan R., Chen H., Lee D.H., Zheng C. (2007). Characteristics of hemicellulose, cellulose and lignin pyrolysis. Fuel.

